# Oral *Fusobacterium nucleatum* exacerbates ulcerative colitis via the oral-gut axis: mechanisms and therapeutic implications

**DOI:** 10.3389/fcimb.2025.1564169

**Published:** 2025-04-07

**Authors:** Zhaoyu Zheng, Wenqin Jin, Weiwei Guo, Zhao Jin, Yuling Zuo

**Affiliations:** ^1^ Chengdu University of Traditional Chinese Medicine, Chengdu, China; ^2^ Hospital of Chengdu University of Traditional Chinese Medicine, Chengdu, China

**Keywords:** *Fusobacterium nucleatum*, oral-gut axis, ulcerative colitis, microbiota, inflammation

## Abstract

**Background:**

*Fusobacterium nucleatum* (*F. nucleatum*) is an anaerobic bacterium known for its association with periodontal disease and oral infections. It has been implicated in the development of gastrointestinal diseases such as inflammatory bowel disease and colorectal cancer. Ulcerative colitis (UC), which is characterized by chronic inflammation of the colon, is a condition of unknown etiology with a rising incidence rate, significantly affecting the quality of life for patients. The increased intestinal permeability during UC may facilitate the adherence or invasion of *F. nucleatum* into the damaged intestinal barrier, leading to exacerbated inflammation.

**Methods:**

This article introduces the concept of the oral-gut axis, reviewing existing literature to analyze the role of *F. nucleatum* in the pathogenesis of UC and exploring its potential pathogenic mechanisms. It also summarizes the latest advances in treating patients with UC who have *F. nucleatum* and looks forward to prospective therapeutic strategies and the translational prospects of *F. nucleatum* within the oral-gut axis.

**Results:**

*F. nucleatum* may be a key player in the pathogenesis of UC, likely due to its invasiveness during periods of increased intestinal permeability. The paper also discusses innovative approaches for the prevention and management of UC exacerbated by *F. nucleatum*, paving the way for more effective treatment of UC.

**Conclusion:**

The review offers new insights into the complex relationship between the oral microbiome and intestinal diseases, enhancing our understanding of their dynamic interactions. There is a paucity of literature on therapeutic approaches, indicating a need for further clinical research.

## 1 Introduction

Fusobacterium nucleatum (*F. nucleatum*), a Gram-negative anaerobic bacterium with a high detection rate in the oral microbiota (Stokowa-Sołtys et al., 2021), is one of the common pathogens responsible for oral microbial dysbiosis prior to the onset of periodontal diseases. It is implicated in the progression of conditions such as periodontitis, gingivitis, and oral squamous cell carcinoma. By adhering to and invading host tissues, *F. nucleatum* promotes inflammation and oral microbial dysbiosis, contributing to the pathogenesis of periodontal diseases due to its strong invasiveness and pro-inflammatory properties (Mesa et al., 2022). *F. nucleatum* acts as a pivotal “bridging” bacterium, facilitating the co-aggregation of oral pathogens and participating in the structural formation of dental plaque biofilms (McIlvanna et al., 2021). The abundance of *F. nucleatum* in the oral cavity is influenced by external environmental factors, such as smoking (Lin P. et al., 2023), systemic glucose levels (Casarin et al., 2013), and poor oral hygiene (Unlu et al., 2024), which alter the oral microenvironment to favor its proliferation and pathogenic effects.

In recent years, *F. nucleatum* has been increasingly linked to extraoral diseases, including adverse pregnancy outcomes (Han, 2015; Vander Haar et al., 2018; Ghosh et al., 2024), atherosclerosis (Figuero et al., 2011; Zhou et al., 2023), and Alzheimer’s disease (Wu et al., 2022). It has virulence mechanisms such as colonization, invasion, induction of host-exacerbated inflammation as well as carcinogenesis (Gholizadeh et al., 2017; Clay et al., 2022). Ulcerative colitis (UC) is a chronic inflammatory disease of the gastrointestinal tract and is classified under the group of Inflammatory Bowel Disease (IBD).The etiology of UC remains unknown, but it is believed that a combination of genetics, environmental factors, immune dysfunction, and diet may contribute to the development of the disease (Gros and Kaplan, 2023). As a result, patients frequently experience symptoms such as abdominal pain, diarrhea, superficial mucosal ulcers, and purulent bloody stools (Mak et al., 2020), which can significantly impair their quality of life. In the past decade, researchers have increasingly focused on the common risk factors for extraintestinal manifestations(EIMs) (Marotto et al., 2020; Rogler et al., 2021) of UC, which can affect various systems including the skin, mucous membranes, eyes, genitourinary system, and musculoskeletal system. Among these manifestations, periodontitis, a prevalent oral disease, has emerged as a significant EIM in patients with IBD (Lam et al., 2023), which is now recognized as one of the most common EIMs associated with IBD. Oral manifestations have been suggested as potential signs of UC (Li et al., 2022)and have been widely explored by scholars. It has been reported that oral symptoms precede some intestinal manifestations in close to 25% of UC patients (Vavricka et al., 2015). Additionally, a case-control study has suggested a higher incidence of oral health problems among patients with IBD than among healthy individuals; however, these oral health issues are rarely effectively addressed (Bertl et al., 2024). The oral cavity, being the uppermost part of the digestive tract, possesses a complex and extensive microenvironment of flora; when the oral cavity is afflicted with disease, the equilibrium of this microbial environment can be disrupted. Consequently, proliferating oral pathogens may traverse the digestive tract and invade the gut, potentially causing an imbalance in the intestinal microbiota and adversely affecting intestinal health (Peng et al., 2022).

Previous research has highlighted that the invasiveness of *F. nucleatum* strains isolated from the inflamed tissues of IBD patients is greater compared to those from normal tissues (Strauss et al., 2011). Moreover, the abundance of these bacteria has been found to correlate positively with the severity and progression of gastrointestinal diseases (Tanwar et al., 2024). Significantly, *F. nucleatum* has been detected in high concentrations in the feces and intestinal tumor tissues of colorectal cancer (CRC) patients (Dregelies et al., 2023; Wang and Fang, 2023). Studies have indicated that *F. nucleatum* may enhance the inflammatory microenvironment that is associated with the progression of CRC (Ternes et al., 2022), and it is also believed to play a crucial role in the development of colitis-associated cancer (CAC) (Jiang et al., 2024). These observations suggest a potential correlation between the oral pathogen *F. nucleatum* and colorectal tumors, but the causal relationship remains unclear. Given that an inflammatory environment is a risk factor for gastrointestinal diseases, and considering that the intestinal tract remains in a state of chronic inflammation during UC, the risk that is of cancer in UC patients is dramatically higher in comparison to healthy individuals (Shah and Itzkowitz, 2022). However, the specific influence of *F. nucleatum* on this risk has not been unequivocally elucidated, and the proposal of the oral-gut axis seems to have made the connection clearer. In this paper, the concept of the oral-gut axis provides insight into the recent developments and prospects of the impact of the oral pathogen *F. nucleatum* on UC disease and suggests targeted therapeutic strategies and challenges.

## 2 The oral-gut axis serves as a “bridge” between *F. nucleatum* and the gut

**Figure 1:**
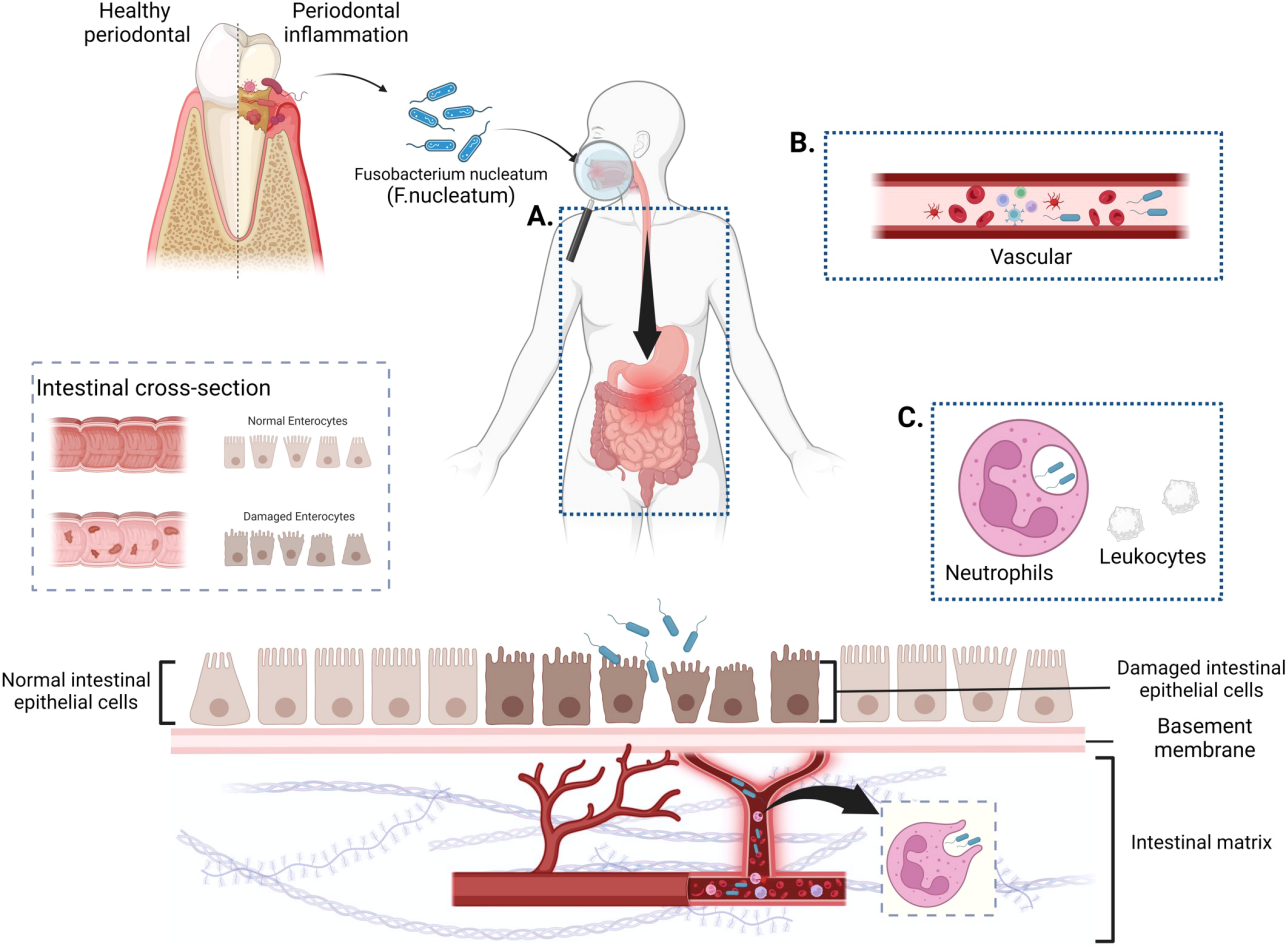
Potential pathways for *F. nucleatum* colonization and invasion of the gut along the oral-gut axis. **(A)**. The digestive tract pathway: *F. nucleatum* enters the gastrointestinal tract through the swallowing of saliva, ingestion of food, or water sources, and establishes intestinal colonization through prolonged oral exposure. **(B)**. Hematogenous transmission route: *F. nucleatum* invades the bloodstream through routine dental activities or oral ulcers and targets the gut for colonization via systemic circulation. **(C)** Indirect pathways (Trojan Horse Hypothesis): Host cells act as a “Trojan Horse” for the hematogenous spread of *F. nucleatum*, facilitating its targeted colonization through this indirect mechanism. (Figure created with BioRender.com).

The oral cavity and gastrointestinal tract serve as the bookends of the digestive system, each hosting intricate and expansive microbial ecosystems. These two realms share components of their microbiota, which are crucial for sustaining health and can significantly contribute to disease pathology. An imbalance in either the oral or gut microbiome can have repercussions for the other, demonstrating that they are intricately linked and collectively impact systemic well-being (Tortora et al., 2023). A diverse oral microbiota, in conjunction with the host immune system, maintains a healthy oral homeostasis, with over 700 species of bacteria known to inhabit the human oral cavity (Xian et al., 2018). When the oral cavity is in a diseased state, the symbiotic balance of the oral flora is disrupted. Low-grade inflammation, such as that found in periodontal disease and caries, can disrupt the natural barriers of the oral cavity or trigger and exacerbate other systemic diseases (Peng et al., 2022). Therefore, the overabundance of certain oral commensal bacteria can incite chronic inflammation by spreading and causing destruction to associated tissues. This discussion outlines three potential pathways through which *F. nucleatum* may colonize and invade the intestinal tract along the oral-gut axis, with the specific illustrations presented in the subsequent figure (**Figure 1**).

### 2.1 The digestive tract pathway

The gut microbiota, akin to the oral microbiota, is viewed as the origin and end places of microbial aggregation in the digestive system, with the presence of gastrointestinal acids, bile acids, barriers in the digestive tract, and resistance to colonization by gut microbes (Kunath et al., 2024) between them, which is considered to be the predominant oral-intestinal barrier. An imbalance of intestinal flora is a prerequisite for the colonization of oral pathogens. If an organism suffers only from oral-related diseases and no intestinal dysbiosis is detected, it is very difficult to have oral pathogen enrichment in the gut. When specific drugs are used or when the gut is continuously exposed to an inflammatory state (Janney et al., 2020; Weersma et al., 2020; Sung et al., 2021), the protective barrier is breached, allowing organisms from the oral microbiota to proliferate and colonize the gut, thereby remodeling the intestinal biota system and progressively exacerbating the intestinal inflammatory microenvironment. Kitamoto et al. (2020), in their investigation of the causal relationship between the oral cavity and the gut, proposed that when both oral and intestinal inflammation are present, oral pathogenic organisms significantly colonize the gut along the digestive tract. This colonization, combined with pre-existing intestinal inflammation, accelerates disease progression due to the ingestion of oral pathogens. Lin S. et al. (2023) verified through mice experiments that *F. nucleatum* colonies only colonized colonic tissues that had been destructively treated with Dextran Sulfate Sodium Salt (DSS).

However, there is also evidence that oral pathogenic bacteria can break the intestinal barrier, that is, following an imbalance in the oral flora, *F. nucleatum* proliferates and enters the digestive tract with saliva and eventually colonises the intestinal surface without the need for inflammation to be involved or flora to be disrupted (Li et al., 2019). Human saliva was transplanted into healthy mice to establish a Human Oral Microbiota in Animals (HOMA) mice model, and experimentally demonstrated that oral flora can overcome the host physical barriers to colonize the intestine, and it was found that *F. nucleatum* belongs to the dominant genus in the intestine, with a relatively high abundance in the small intestine and distal intestinal tract. Thus, it was demonstrated that passing through the digestive route only proved a bi-directional association between oral flora and UC, but how it is affected remains unclear.

### 2.2 Hematogenous transmission route

As a result of tissue trauma from dental surgery (Rajasuo et al., 2004) or disruption of the gingiva or periodontium due to chewing or mechanical factors (Lockhart et al., 2008), oral flora can spread via the hematogenous route through vesicular or ulcerated surfaces into the body’s circulation and eventually colonize target tissues. The occurrence of bacteremia is more likely in patients with inadequate oral hygiene (Forner et al., 2006). Although the mechanism of bacteremia is not fully understood, it has been experimentally demonstrated (Tsukasaki et al., 2018) that when the oral barrier is compromised, oral bacteria can disseminate along the systemic circulation, affecting overall health.

When transient bacteria enter the bloodstream, the body’s immune system recognizes the infection and clears the infection, preventing systemic circulation transmission. However, in cases of severe periodontal disease or chronic inflammatory disease, the inflammatory response disrupts the tight junctions between endothelial cells, thereby increasing vascular permeability and permitting bacteria and other immune cells to enter the vessel wall and surrounding tissues, further accelerating the progression of inflammation (Nakajima et al., 2010). FadA, a surface adhesin expressed by *F. nucleatum* (described in further detail below), can bind to vascular endothelial-cadherin (VE-cadherin), increasing tissue permeability and altering endothelial integrity. This contributes to the penetration of *F. nucleatum* into endothelial cells (Pignatelli et al., 2023), suggesting that hematogenous transmission may be a pathway for oral bacteria to enter the body’s circulation and spread to target organs. Given that *F. nucleatum*, a common oral pathogen, exhibits high abundance in periodontal disease patients, its potential risk of hematogenous dissemination warrants attention. Currently, scant clinical evidence exists in this field, necessitating further evidence-based medical research to validate this hypothesis.

### 2.3 Indirect pathways

In recent years, some scholars have proposed the Trojan horse hypothesis (Moulder, 1975), which is a theory surrounding the role of innate immune cells in transporting and dispersing pathogens. For instance, dendritic cells can transport *Porphyromonas* (*P. gingivalis*), a bacterium found in the gums of patients with periodontal disease, from the oral cavity to the arterial walls (Carrion et al., 2012). Host cells serve as an indirect means of bacterial spread through the bloodstream, acting as “Trojan horses.” (Hajishengallis, 2015)Felix Ellett (Ellett et al., 2023) and colleagues experimentally confirmed the indirect pathway of *F. nucleatum* spreading within the body in accordance with this hypothesis. Their experiments demonstrated that *F. nucleatum* can survive within neutrophils after phagocytosis and spread within a zebrafish experimental model with the involvement of white blood cells, supporting the microbial transmission hypothesis linking oral diseases with certain systemic conditions. However, more evidence is needed to fully describe the indirect transition of *F. nucleatum* from an oral pathogen to the gut.

## 3 Examination of *F. nucleatum*-Mediated Inflammation in UC

### 3.1 Animal models that can be used to explore the mechanism of *F. nucleatum* inflammation in UC disease

**Table 1** provides a main summary of animal models used to explore the inflammatory mechanisms of *F. nucleatum* in UC disease, with detailed information found in the table.The animal models currently used to investigate the mechanisms of *F. nucleatum*-mediated inflammation in UC are closely related to the potential modes of colonization and transmission described in the previous section. For example, the digestive tract pathway is simulated by administering *F. nucleatum* via oral gavage, which mimics the natural route of oral bacteria entering the gut through ingestion. Similarly, models that induce periodontal disease to promote bacterial dissemination indirectly reflect the hematogenous transmission route, demonstrating how *F. nucleatum* can enter systemic circulation. These models collectively provide a comprehensive framework for understanding how *F. nucleatum* colonizes and exacerbates inflammation in UC through various transmission pathways.

**Table 1:** Summary of animal models exploring the inflammatory mechanisms of *F. nucleatum* in UC.

Animal models used	Selection of *F. nucleatum*	*F. nucleatum’s* methods of colonization	Selection of DSS concentration	Methods	Main Findings	References
C57BL/6J mice (Males,6 weeks)	ATCC25586 1×10^9^ CFU/mL	Oral method	2.5% DSS solution	The mice in the model group were given 2.5% DSS solution as drinking water for six consecutive days, followed by six days of distilled water, completing three cycles in total. During this period, the mice were orally administered *F. nucleatum* suspension every three days, with a dose of 10 mg/kg.	*F. nucleatum* promoted alveolar bone loss and colonized only infected colonic tissues, further exacerbating intestinal inflammation and epithelial barrier damage.	Lin et al. (2023)
C57BL/6mice (Males,8 weeks)	FnEVs 1×10^9^ CFU/mL	Oral method	3% DSS solution	In the FnEVs-treated group, FnEVs were administered every day by gavage to each mouse. The modeling group was given a 3% DSS solution mixed with drinking water for seven days to establish colitis. The modeling cycle was ten days.	FnEVs exacerbates DSS-induced acute colitis by increasing intestinal barrier permeability and attenuating intestinal barrier function through RIPK1-mediated apoptosis.	Liu et al. (2021)
C57BL/6J wild-type mice(Males,6–8 weeks)	ATCC25586 1×10^8^ CFU/mL	Gavage	3% DSS solution	Exosomes purified from ATCC25586 and serum exosomes extracted from humans and mice were injected into the tail vein at a dosage of 50 μg per mouse every two days. This injection regimen started one day before the mice received DSS treatment, which was administered for seven days.	Exosomes delivered miR-129-2-3p from *F. nucleatum*-infected IECs into non-infected IECs, exacerbating epithelial barrier dysfunction and experimental colitis	Wei et al. (2023a)
C57BL/6 mice (Males,6-8 weeks)	ATCC25586 1×10^9^ CFU/mL	Gavage	2.5% DSS solution	Oral administration of 2 mg/ml streptomycin for 3 days before the experiment promoted colonization. Colitis was induced in mice by tube feeding with daily administration of *F. nucleatum* suspension for 2 weeks, followed by 2.5% DSS for 7 days.	*F. nucleatum* is abundant in UC tissues and correlates with clinical features, and its LPS exacerbates IEC death by promoting IEC autophagy. Therefore, *F. nucleatum* may contribute to UC by activating autophagic cell death.	Su et al. (2020)
C57BL/6 mice (Females,6 weeks)	ATCC25586 1×10^9^ CFU/mL	Gavage	2.5% DSS solution	Mice received a single intraperitoneal injection of azoxymethane at a dose of 10 mg/kg. One week later, the mice were given 2.5% DSS in their drinking water for 7 days, followed by 14 days of normal drinking water for recovery. This cycle was repeated three times. F. nucleatum was administered by gavage six times until the end of the 12-week study period.	*F. nucleatum* accelerates the progression of CAC by promoting EMT through the EGFR signaling pathway.	Yu et al. (2020)
BABL/c mice (Males,6-8 weeks)	Feces from patients with UC 1×10^9^ CFU/mL	Gavage	5% DSS solution	Mice receiving bacteria were gavaged with 500 ul of bacterial solution daily for one week. One week after infection, colitis was induced by water-mediated administration of 5% DSS, which was allowed to be consumed ad libitum by mice for 7 days.	*F. nucleatum* can promote the progression of UC via proinflammatory M1 macrophage skewing, and targeting F. nucleatum or AKT2 signaling may be a viable means of blocking the development of UC.	Liu et al. (2019)
Humanized microbiota C57BL/6 mice(Males,10-16 weeks)	*Fusobacterium nucleatum subspecies polymorphum(F. nucleatum subspecies polymorphum)* ATCC 10953 1×10^9^ CFU/mL	Gavage	/	Mice were administered an antibiotic cocktail, metronidazole, and vancomycin ad libitum in drinking water for 3 to 5 days. After 24 hours, they were injected with clindamycin. Then, 24 hours later, they were orally administered *F. nucleatum subspecies polymorphum* in PBS.	*F. nucleatum subspecies polymorphum* adheres to intestinal mucus and secretes OMVs. *F. nucleatum subspecies polymorphum* secreted compounds and purified OMVs promote the secretion of colonic proinflammatory cytokines. *F. nucleatum* can promote inflammation in normal epithelial cells *in vitro* and *in vivo*.	Engevik et al. (2021)
C57BL/6 mice (Males,6 weeks)	ATCC25586 1×10^9^ CFU/mL	Gavage	2.5% DSS solution	After 2 weeks of oral antibiotic administration to mice, mice were given *F. nucleatum* twice a week by gavage. One week later, colitis was induced by three cycles of 2.5% DSS (7 days of DSS induction followed by 14 days of water).	*F. nucleatum* was significantly enriched in the feces of IBD patients and its abundance correlated with disease activity. *F. nucleatum* disrupts epithelial integrity and increases permeability by regulating the expression and distribution of the tight junction proteins zonula occludens-1 and occludin.	Liu et al. (2020)
C57BL/6 mice (Males,6 weeks)	ATCC25586 1×10^9^ CFU/mL	Gavage	3% DSS solution	Mice in the model group were given *F. nucleatum* for seven days and then 3% DSS orally for seven days to induce colitis.	*L. rhamnosus* plays a protective role in the pathogenesis of *F. nucleatum*-related colitis and that the mediation of autophagy is involved in this process.	Duan et al. (2023)
C57BL/6 mice (Females,6weeks), TLR2 and TLR4 mice	ATCC25586 1×10^8^ CFU/mL	Oral method	\	Mice were orally administered *F. nucleatum* for one week, and all mice were sacrificed after two months.	TLR2/TLR4 regulate *F. nucleatum*-induced inflammatory cytokines through Tregs *in vivo*.	Jia et al. (2017)
C57BL/6J wild-type (CARD3wt) mice (males,6 weeks), C57BL/6J CARD3 knockout(KO, CARD3–/–)mice(Males,5-6weeks)	From UC patients 1×10^9^ CFU/mL	Gavage	3% DSS solution	All mice were given streptomycin in the drinking water for 3 days. The mice were then given *F. nucleatum* by gavage for 2 weeks, followed by ad libitum consumption of 3% DSS to induce colitis.	*F. nucleatum* was enriched in 51.78% of UC tissues and was correlated with the clinical course, clinical activity, and refractory behavior of UC.	Chen et al. (2020)
BABL/c mice (Males, 6 weeks)	ATCC25586 1×10^9^ CFU/mL	Oral method	5% DSS solution	Mice were orally administered *F. nucleatum* daily for one week and then given 5% DSS ad libitum for one week to induce colitis.	*F. nucleatum* infection facilitates inflammation in acute colitis with IL-1α from colon tissue by activating noncanonical inflammasome through gasdermin D cleavage.	Boonyaleka et al. (2023)
C57BL/6 mice (Males,6 weeks)	ATCC25586 1×10^9^ CFU/mL	Gavage	3% DSS solution	Mice in the model group were given *F. nucleatum* for seven days and then 3% DSS orally for seven days to induce colitis.	Fucose can enhance the inflammatory properties of *F. nucleatum* by modifying its metabolism, indicating the potential use of fucose as a functional food or prebiotic in treating *F. nucleatum*-related colitis.	Duan et al. (2021)
C57BL/6 mice (Males,6 weeks)	Strain extracted from feces of CRC patients 1×10^9^ CFU/mL	Gavage	3% DSS solution	Self-administered drinking with 3% DSS solution was used to establish a mice colitis model. Gavage using *F. nucleatum* was administered 2 weeks before establishing the model and an intraperitoneal injection of norepinephrine was performed thirty minutes later.	The NE-QseC axis enhances the pathogenicity of *F. nucleatum* through interkingdom signaling, aggravating colonic inflammation in IBD mice. QseC could be a potential target for microbiota management of IBD under chronic pressure.	Zhang et al. (2024a)
C57BL/6 mice (Males,6 weeks)	ATCC25586, fadA gene-harbouring ATCC 25,586, ATCC 12,230-US1, 1×10^9^ CFU/mL	Brushing the oral cavity with *F. nucleatum* daily	2.5% DSS solution	Drinking water supplemented with 2 mg/ml streptomycin was given to each group of mice for three days to promote *F. nucleatum* colonization before induction of colitis. After this, 2.5% DSS was administered for 14 days, and the oral cavity was brushed daily with the corresponding strain solution for each group to ensure complete coverage of the oral cavity.	Oral inoculation with *F. nucleatum* facilitates experimental colitis via the secretion of the virulence adhesin FadA.	Li D. et al. (2024)
C57BL/6J mice (Males, 6-8 weeks)	ATCC25586 1×10^9^ CFU/mL	Oral method	2.5% DSS solution	Drinking water supplemented with 2 mg/ml streptomycin was given to each group of mice for 3 days to promote *F. nucleatum* colonization before induction of colitis. After that, 2.5% DSS was given for 14 days and the mice were orally inoculated with *F. nucleatum* daily.	Oral incubation of *F. nucleatum* further exacerbates the severity and dysbiosis in DSS-induced colitis mice. Besides, lower tract FMT can ameliorate colitis by restoring the gut microbiota diversity and eliminating *F. nucleatum* and virulence factor fadA.	Li DH. et al. (2024)
C57BL/6N mice(Females,6 weeks)	ATCC25586 1×10^10^ CFU/mL	Oral method	2% DSS solution	Mice were orally administered *F. nucleatum* 27 days. 3 days later, 2% DSS was given to induce colitis and continued for 8 days. Normal water intake was then given for 8 days to reduce intestinal inflammation.	*F. nucleatum* hindered the mice’s recovery from DSS-induced colitis and prolonged their colon inflammation, likely because it altered the MAM compositions.	Yamashita et al. (2021)

This table provides a comprehensive overview of the selection of experimental animals, strains of *F. nucleatum*, DSS concentrations, specific experimental methods, and key research findings. It appears that most studies investigating the inflammatory mechanisms of *F. nucleatum* in UC utilize murine co-morbidity models. Depending on the research focus, various strains or fractions of *F. nucleatum* are selected, and the DSS concentration is not standardized.

UC, Ulcerative Colitis; CAC, Colitis-associated cancer; EMT, Epithelial-mesenchymal transition; *L. rhamnosus*, *Lactobacillus rhamnosus*; NE, norepinephrine; QseC, Quorum sensing regulators C; FMT, Fecal Microbiota Transplantation; CRC, colorectal cancer; DSS, dextran sulfate sodium; MAM, Mucosa-associated microbiota; FnEVs(consist of *F. nucleatum* shedding bacterial outer membrane and periplasmic components.); IEC, intestinal epithelial cell; LPSlipopolysaccharide; OMVs, Outer membrane vesicles.

Currently, most researchers exploring the profound effects of *F. nucleatum* in UC utilize mouse models, with c57BL/6 and BABL/c mice being widely preferred by scholars. Studies investigating the inflammatory mechanisms of *F. nucleatum* in gastrointestinal diseases such as UC and CRC, primarily rely on co-morbid models. To simulate the inflammatory environment of UC disease in experimental animals, DSS solution is widely used due to its pathological similarities with human UC (Lin S. et al., 2023) (Su et al., 2020; Yu et al., 2020; Liu et al., 2021; Wei et al., 2023a). Additionally, oral pathogenic bacteria *F. nucleatum* are administered via gavage or induced through periodontal disease in animals to provide a continuous supply of the pathogen. Specific experimental procedures are recommended to vary slightly among different researchers.

As reported, UC disease modeling with DSS typically takes about 6-7 days, and the concentration of DSS used ranges from 2.5% to 5%, which was added to the water according to the desired concentration for the experimental animals to consume freely (Liu et al., 2019; Duan et al., 2023) (Liu et al., 2020). *F. nucleatum* has been selected by some scholars to culture the strain (ATCC25586) (Su et al., 2020; Yu et al., 2020; Wei et al., 2023a), and some scholars have chosen to use the feces of UC patients to extract *F. nucleatum* and then enter the experimental animals by gavage feeding method (Liu et al., 2019). Most scholars observed corresponding indexes after *F. nucleatum* gavage at the end of UC modeling, while others carried out UC modeling before (Jia et al., 2017; Chen et al., 2020; Duan et al., 2021; Engevik et al., 2021; Yamashita et al., 2021; Boonyaleka et al., 2023; Duan et al., 2023). Yu et al. (2020) and others have deviated from previous methodologies by adopting the method of alternating administration of *F. nucleatum* at weeks 2, 5, and 8 of the 10-week experimental period. Some scholars administered antibiotics such as streptomycin and vancomycin before the start of the experiment to reduce the native gut microbiota, thereby creating a more favorable environment for *F. nucleatum* colonization (Chen et al., 2020; Liu et al., 2020; Li DH. et al., 2024).

### 3.2 Multifaceted virulence mechanisms of *F. nucleatum* in UC

#### 3.2.1 Intestinal epithelial barrier disruption and inflammation activation

*F. nucleatum* damages the intestinal epithelial barrier by disrupting the connections between the cells (Liu et al., 2020). It has been observed that TLR2/TLR4 regulated *F. nucleatum in vivo* to induce inflammatory cytokines through Tregs and modulate intestinal inflammation when only *F. nucleatum* intervened without DSS to induce intestinal inflammation (Jia et al., 2017). Chen et al. (2020) discovered that *F. nucleatum* was enriched in patients with UC, and it was found to be correlated with the severity of the condition. Furthermore, it was experimentally demonstrated that the abundance of *F. nucleatum* could be regulated through the activation of Caspase recruitment domain3 (CARD3), the *F. nucleatum* infection further exacerbates epithelial damage, promotes the expression of inflammatory cytokines IL-1β, IL-6, IL-17F, and TNF-α, and promotes intestinal inflammation *in vivo* and *in vitro* via the IL-17F pathway, which plays a key role in mediating UC development through upregulation of CARD3 and activation of the IL-17F/NF-κB classical inflammatory pathway. Macrophages likewise play a vital role in regulating the intestinal mucosal barrier. *F. nucleatum* can induce macrophage mobilization, promoting their bias towards the M1 phenotype through the AKT2 signaling pathway. Subsequently, the release of massive inflammatory cytokines further impairs the intestinal mucosal barrier, resulting in the localization of bacteria, which leads to the development and progression of UC (Liu et al., 2019).

#### 3.2.2 Metabolic products and immunomodulatory imbalance

During the growth of Fusobacterium nucleatum, the primary chemotactic factors released are short-chain fatty acids (SCFAs), predominantly acetate and butyrate (Dahlstrand Rudin et al., 2021). While *F. nucleatum* produces significant amounts of butyrate, which is a crucial anti-inflammatory component in colonic tissues (Stokowa-Sołtys et al., 2021), this contrasts with its role in the progression of UC. Studies have revealed that butyrate serves as the preferred energy source for colonic mucosal cells, and insufficient energy supply to these cells may contribute to the pathogenesis of colonic inflammation (Pryde et al., 2002).Although butyrate produced by *F. nucleatum* serves as the primary energy source for colonic mucosal cells, its pro-inflammatory role in UC necessitates a reevaluation of the applicability of butyrate supplementation therapy. In the future, personalized nutritional interventions may be developed by detecting the abundance of *F. nucleatum* and butyrate levels in patients’ intestines, such as supplementing specific probiotics to promote butyrate production or restricting carbon sources readily utilized by *F. nucleatum*.

Lipopolysaccharide (LPS), an outer cell wall component shed when *F. nucleatum* dies and lyses, is a pathogenic virulence factor. LPS drives the autophagic activation of intestinal cells, induces autophagy and exacerbates autophagic death in intestinal epithelial cells (IECs) both *in vivo* and *in vitro*, thereby promoting UC progression through this specific autophagic pathway (Su et al., 2020); Boonyaleka et al. (2023) experimentally verified that LPS activates caspase-11 in macrophages, induces pyroptosis during infection, activates atypical inflammatory vesicles via Gasdermin D (GSDMD) cleavage, and thus promotes inflammation in acute colitis by utilizing IL-1α in the colonic tissue.Defective autophagy may significantly influence the progression of IBD by disrupting intestinal homeostasis, altering the microbiota, impeding bacterial clearance, and exacerbating inflammation (Larabi et al., 2020). LPS-induced autophagic death of IECs suggests that inhibition of excessive autophagy could serve as a therapeutic strategy.

#### 3.2.3 Indirect damage by OMVs and FnEVs

Outer membrane vesicles (OMVs), which are spontaneously secreted in a programmed manner on the outer cell membrane of *F. nucleatum*, play a crucial role in the *F. nucleatum*-mediated inflammatory response. At this point, *F. nucleatum* interacts with the host indirectly and promotes pro-inflammatory effects by activating TLR4 and downstream targets ERK, CREB, and NF-κB, thereby inducing the production of cytokines that cause inflammation. Notably, it was demonstrated in mice colonized with human gut microbiota that healthy gut barriers are resistant to adverse effects from OMV secreted by *F. nucleatum* (Engevik et al., 2021).The resistance of healthy gut barriers to OMVs suggests that the fecal OMV level may serve as a biomarker for mucosal healing. In combination with endoscopic examination, it could potentially be used to evaluate the treatment response in patients with UC.

F. *nucleatum* secretes extracellular vesicles (FnEVs) in the gut carrying a variety of deleterious molecules that alter microbe-host interactions, which significantly inhibit levels of ZO-1, claudin-1 and occludin, disrupt colonic tissue structure and increase DSS administration-induced intestinal damage and intestinal barrier permeability, while FnEVs promote macrophage polarisation to a pro-inflammatory phenotype, induce oxidative damage to cells, and later induce pro-inflammatory macrophages, promote epithelial cell apoptosis, which in turn attenuates intestinal barrier function, promotes pro-inflammatory macrophage differentiation and accelerates IEC necrotic apoptosis through activation of the FADD-RIPK1-caspase3 signaling pathway, and ultimately exacerbates intestinal barrier damage (Liu et al., 2021); Wei et al. (2023b) also noted the barrier disruption of the intestine by FnEVs, suggesting that experimental colitis in mice was significantly worsened by FnEVs, which caused down-regulation of miR-574-5p expression and activation of autophagy.Therefore, targeting FnEVs may emerge as a novel strategy for intestinal barrier repair, which warrants further investigation by scholars.

Exosomes within extracellular vesicles are likewise one of the research focuses in the industry, which deliver miR-129-2-3p from *F. nucleatum*-infected IECs to uninfected IECs, and were further uncovered to explore the role of *F. nucleatum*-Exo, a *F. nucleatum*-infected epithelial cell-derived exosome, in the induction of DNA via the miR-129-2-3p/TIMELESS axis induces DNA damage and later activates the ATM/ATR/p53 pathway, thereby promoting cellular senescence, exacerbating intestinal barrier damage and experimental colitis (Wei et al., 2023a).

#### 3.2.4 Adhesin-mediated colonization and invasion

As an adherent bacterium, *F. nucleatum* produces an adhesin called FadA, which attaches to host cells. FadA is the most extensively studied virulence factor (Clay et al., 2022), affecting E-cadherin, β-catenin proteins that are tightly linked in intestinal epithelial cells, and subsequently disrupting the intestinal barrier (Guo et al., 2020). A study demonstrated that individuals with adenomas and adenocarcinomas have higher levels of the FadA gene in their colon tissues compared to healthy individuals, suggesting that FadA may be a key factor in gastrointestinal tumor diseases (Rubinstein et al., 2013). The specific high expression of the FadA gene in adenoma and adenocarcinoma tissues suggests its potential as a molecular biomarker for early screening of CRC. By integrating fecal microbial detection or blood-based cell-free DNA analysis, non-invasive diagnostic tools could be developed. Meng et al. (2021) found that *F. nucleatum* secretes amyloid-like FadA through a Fap2-like autotransporter protein to promote *F. nucleatum* colonization *in vivo* and affect CRC progression. The virulence gene FadA carried by *F. nucleatum* may serve as a potential pathogenic and exacerbating factor in UC. When orally commensal *F. nucleatum* translocates to the gastrointestinal tract under pathological conditions, it adheres to and invades epithelial cells via the FadA adhesin, triggering the secretion of pro-inflammatory cytokines. This process further induces intestinal epithelial inflammation, contributing to the progression of UC. However, the precise mechanisms underlying these effects remain incompletely elucidated (Li et al., 2021).

In addition, *F. nucleatum* possesses another outer membrane protein, Fibroblast Activation Protein 2 (Fap2). During CRC, *F. nucleatum* colonizes colorectal tumor tissues via a hematogenous route in a Fap2-dependent manner (Abed et al., 2016). Through intravascular injection, Fap2 binds to acetylgalactosamine (Gal-GalNAc), thereby contributing to CRC progression. In the mucus layer of colonic tissues, the aggregation and adhesion of *F. nucleatum* with Clostridioides difficile (C. difficile) are facilitated by the adhesin RadD (Luna et al., 2021). Aggregation is the initial step in biofilm formation and a critical factor for bacterial colonization. RadD promotes the colonization and enrichment of *F. nucleatum* on CRC cells, accelerating disease progression (Zhang et al., 2024b). An increased abundance of FadA/Fap2 genes in the gut microbiota of UC patients may indicate a predisposition to carcinogenesis. Therefore, it is recommended to regularly monitor the expression of F. nucleatum virulence genes in long-term UC patients, combined with colonoscopic biopsies for risk stratification.

Currently, the evidence supporting the virulence mechanisms of FadA, Fap2, and RadD primarily stems from their effects on intestinal inflammation and tumor cells in CRC. Given the overlapping microbiota between IBD and CRC (Li et al., 2023), eradicating F. nucleatum colonization may confer dual benefits. However, further research is needed to predict and validate the roles of these adhesins in the virulence mechanisms of UC.

#### 3.2.5 Neurotransmitters and stress effects

Norepinephrine (NE) binds to the group sensing regulator QseC of *F. nucleatum*, enhancing its pathogenicity, including virulence and invasiveness. This interaction subsequently disrupts the intestinal barrier and exacerbates UC progression. In stressful situations, NE levels in the gut are tens of times higher than under normal conditions, implying that long-term chronic stress may also be an aggravating factor for UC (Zhang et al., 2024a).

At present, medical experts are uncertain whether *F. nucleatum* is responsible for causing UC, a type of intestinal inflammation. Although some studies have demonstrated that *F. nucleatum* can have adverse effects on intestinal inflammation, the root cause of UC is still under investigation. It is imperative to conduct further research to understand the mechanisms by which *F. nucleatum* might be responsible for triggering UC, in order to find ways to prevent its progression.

## 4 Therapeutic strategies for targeting *F. nucleatum* in UC

### 4.1 Controversies and optimized applications of antibiotics

The role of antibiotics in the therapeutic management of UC remains controversial. The use of broad-spectrum antibiotics has been shown to reduce fecal microbial diversity in patients, with effects lasting up to six months (Dethlefsen et al., 2008). Some researchers have also suggested that antibiotic use may alter the overall community structure of the gut microbiota in patients with in IBD, potentially exacerbating dysbiosis and increasing the risk of disease progression (Gevers et al., 2014). In UC treatment, broad-spectrum antibiotics, such as metronidazole and ciprofloxacin, are empirically used to reduce bacterial load and alleviate acute inflammation (Glassner et al., 2020). Notably, metronidazole has been shown to effectively reduce the abundance of *F. nucleatum* in CRC (Jiang et al., 2023). However, the long-term efficacy and safety of antibiotic-based therapeutic strategies in UC require further validation in clinical settings. Indiscriminate use of antibiotics may disrupt commensal microbiota, exacerbate dysbiosis, and inadvertently promote the growth and colonization of pathogenic bacteria, including *F. nucleatum* Targeted antibiotic regimens, combined with probiotics or microbiota restoration therapies, may help mitigate these risks.

### 4.2 5-ASA therapy and microbiota-targeted combination strategies

Currently, the primary treatment for mild-to-moderate UC involves 5-aminosalicylic acid (5-ASA) derivatives, including sulfasalazine, mesalamine, and diazo-bonded 5-ASA formulations (Ko et al., 2019). However, these therapies primarily address host inflammatory responses rather than underlying microbial dysbiosis. Studies have shown that *F. nucleatum* may contribute to chemoresistance by modulating the tumor microenvironment or activating pro-survival pathways in epithelial cells, thereby reducing treatment efficacy (Zhang et al., 2019). This highlights the necessity of adopting combinatorial approaches that simultaneously target inflammation and pathogenic microbiota. Future therapeutic strategies may integrate anti-inflammatory drugs with *F. nucleatum*-specific interventions to break the vicious cycle of chronic inflammation and microbial persistence.

### 4.3 Potential and mechanisms of FMT

Fecal Microbiota Transplantation (FMT) is a biological therapy that involves transferring functional microbial communities from a healthy individual’s feces into a patient’s gastrointestinal tract, aiming to restore the gut microbiome to treat diseases. Initially used for the treatment of refractory *Clostridioides difficile* (*C. diff*), FMT has shown significant clinical efficacy (Minkoff et al., 2023). Numerous studies have now demonstrated that FMT can contribute to the alleviation of UC (Fang et al., 2021; Huang et al., 2022; Lahtinen et al., 2023). Further animal experiments have demonstrated that the transplantation of fecal microbiota from the lower gastrointestinal tract can not only restore the disrupted gut microbiome in mice with dextran sulfate sodium (DSS)-induced colitis but also decrease the levels of *F. nucleatum* and the virulence factor fadA (Li DH. et al., 2024). However, limited data exist regarding FMT targeting the growth regulation of *F. nucleatum*. Relevant scholars have investigated whether fecal microRNAs (miRNAs) influence gut microbial communities, and discovered that fecal miRNAs can specifically target genes of *F. nucleatum* and Escherichia coli (*E. coli*), thereby modulating the gut microbiota (Liu et al., 2016). These findings reveal host defense mechanisms and establish a foundation for further exploration of their therapeutic potential. Animal experiments demonstrated that transplantation of fecal microbiota from the lower gastrointestinal tract ameliorated gut dysbiosis in DSS-induced colitis mice while reducing *F. nucleatum* abundance and fadA virulence factor levels (Li DH. et al., 2024). This provides novel insights into understanding the oral-gut axis and the potential use of specific oral-associated bacteria as biomarkers for predicting FMT therapeutic efficacy, which warrants further in-depth exploration.

### 4.4 Probiotic and prebiotic interventions

Probiotic supplementation in patients with IBD offers new avenues for disease treatment and intervention due to its safety, efficacy, and good tolerability (Marchesi et al., 2016). The probiotic *Lactobacillus rhamnosus* (*L. rhamnosus*) has been shown to alleviate colitis exacerbated by *F. nucleatum* in a model, protecting the intestinal tract through the reduction of pro-inflammatory factors and the activation of autophagy (Duan et al., 2021). Kefir, a fermented milk drink originating from Tibet, is typically made with cow’s, sheep’s, or goat’s milk. It is fermented with kefir grains that contain lactic acid bacteria and yeast. Compared to regular yogurt, kefir has superior beneficial effects on the digestive tract due to its probiotic population and fermentation method (Azizi et al., 2021). A previous study has demonstrated that kefir can reduce inflammation and protect the colonic barrier (Zeng et al., 2021). Screening has revealed that one of these probiotics, *Saccharomyces cerevisiae JKSP39* (*S. cerevisiae JKSP39*, *SC*), exhibits the best probiotic properties *in vitro*. It could improve colitis induced by *F. nucleatum*-DSS by reducing reactive oxygen species in colonic tissues and inhibiting endoplasmic reticulum stress, thereby achieving a coloprotective effect through the regulation of the intestinal microbiota (Zeng et al., 2022). Further studies on Kefir have revealed that the Kefir supernatant, which belongs to sterile whey, can improve the symptoms of colitis induced by *F. nucleatum*-DSS. This improvement is achieved by inhibiting the secretion of pro-inflammatory cytokines such as TNF-α, IL-6, and IL-17F, facilitating the release of anti-inflammatory cytokines like IL-4 and IL-10, and ameliorating oxidative stress to restore the intestinal microbial community structure (Zeng et al., 2024). Thus, Kefir could be further explored as a potential probiotic in the treatment against *F. nucleatum*. Fucoidan has been suggested to play a role in maintaining intestinal homeostasis as a prebiotic in recent years (Goto et al., 2016), and Duan et al. (2023) in a further study found that fucoidan improved the pro-inflammatory properties of *F. nucleatum* in colitis, where it altered the metabolism and reduced the production of pro-inflammatory metabolites, which resulted in a reduction of apoptosis, autophagy blockade of IECs and damage to intestinal epithelial tight junctions.

### 4.5 Exploration of vaccines and immunotherapy

Emerging therapeutic strategies targeting *F. nucleatum* have expanded beyond conventional approaches. Notably, the development of vaccines against *F. nucleatum* holds significant therapeutic promise. As a mediator of tumor promotion and treatment resistance, *F. nucleatum* offers unique and unconventional opportunities for vaccine-based interventions. In the context of periodontal disease treatment, DNA vaccines encoding *F. nucleatum* outer membrane proteins, such as FomA, have demonstrated immunogenicity in animal studies, inducing specific antibody responses that inhibit bacterial adhesion (Liu et al., 2010). Although the development of *F. nucleatum* vaccines for antibacterial, anti-inflammatory, and antitumor applications remains a lengthy and complex process, researchers have identified key adhesins, including Fap2, RadD, and FadA, as candidate antigens for subunit vaccines (Holt, 2023). These adhesins are hypothesized to elicit antibodies that block tumor-promoting functions. Although clinical trials have yet to be initiated, these findings underscore the potential of immunotherapy in mitigating *F. nucleatum*-associated pathologies.

### 4.6 Oral health interventions and axis disruption strategies

Existing studies have delved deeply into the regulation of the gut microbiome, yet there are few therapeutic strategies specifically targeting *F. nucleatum* in UC. Consequently, it is too early to claim that treatments targeting *F. nucleatum* for UC are well-developed. Currently, a cure for UC remains elusive, and the main objectives of therapy are to achieve clinical remission and endoscopic healing (Le Berre et al., 2023). A plethora of research has highlighted the intimate link between gut microbiome dysbiosis and intestinal inflammation (Cai et al., 2022). This inflammation is primarily due to the interplay of host genetic and environmental factors, which result in a shift in the host’s immune response to the gut microbiota (Fan et al., 2022). Environmental factors, including diet and lifestyle, can disrupt the balance of the gut microbiota, leading to issues such as microbial translocation, diminished biodiversity, and the instability of microbial colonies in inflammatory conditions (Wang A. et al., 2024). The oral microbiome functions like a reservoir, ceaselessly funneling potential pathogens from the oral environment to the gut via the oral-gut axis, thereby perpetuating intestinal inflammation. Based on this, we hypothesize that improving the oral microbial environment, with a particular focus on treating oral diseases in UC patients, and mitigating the downstream effects of oral pathogens on the gut, may represent a promising therapeutic target. This approach may help in reducing the exacerbation of intestinal inflammation associated with UC.

To complement systemic interventions, reducing the abundance of *F. nucleatum* in the oral cavity may serve as a preventive measure to inhibit its translocation to the gut. For instance, the supernatant from Lactobacillus reuteri AN417 cultures (LRS) has been shown to significantly compromise biofilm integrity and exhibit antimicrobial activity against periodontal pathogens, including *F. nucleatum* (Yang et al., 2021). Additionally, the application of antimicrobial peptides (AMPs) has demonstrated efficacy in inhibiting biofilm formation by *F. nucleatum* and Porphyromonas gingivalis (Matsugishi et al., 2021). These approaches represent promising strategies to disrupt the oral-gut axis transmission and warrant further investigation.

### 4.7 Integrative treatment strategies and future perspectives

Ranging from initial case reports to subsequent cross-sectional studies, a substantial body of research has highlighted a significant link between periodontal disease and IBD (Lauritano et al., 2019; Baima et al., 2023; Wang Q. et al., 2024). Despite this, direct evidence linking the treatment of UC to the outcomes of periodontal disease remains scarce. Nonetheless, certain discoveries lend credence to this association. In pertinent mice models, periodontitis treated with exosomes derived from mesenchymal stem cells has been shown to reduce the severity of experimental colitis (Zhang et al., 2021). Furthermore, a clinical study has demonstrated that IBD patients undergoing treatment with anti-tumor necrosis factor-alpha biologic medications exhibit an adjunctive therapeutic effect on their apical periodontitis (Cotti et al., 2018). These findings reiterate the significance of an integrated oral and intestinal treatment strategy in managing UC.

*F. nucleatum*, a prevalent oral pathogen, poses a significant risk to the gastrointestinal system. The potential of oral disease treatment to mitigate the accelerated colonization and invasion of *F. nucleatum* along the oral-gut axis, and consequently delay the progression of UC, is a concept that warrants further investigation by scholars in the field. In addition, fresh fruits and vegetables have been shown to affect gastrointestinal health positively. Cellular experiments have demonstrated that the extracellular vesicles from tomatoes can inhibit *F. nucleatum* in the intestinal tract, highlighting their potential as a natural source of lipid-mediated antimicrobial agents (Lee et al., 2023). Furthermore, innovative therapeutic strategies derived from food sources could also be potentially exploitable.

## 5 Summary

UC often results in a prolonged condition due to chronic inflammation, intestinal microecological dysregulation, and intestinal barrier disruption, severely impacting patients’ quality of life. *F. nucleatum*, a common oral pathogen, has been linked to adverse effects in systemic diseases according to numerous studies. In this paper, we examine the oral-gut axis, detailing the various pathways through which *F. nucleatum* spreads within the host and emphasizing its inflammatory mechanisms. We conclude with an outlook on potential therapeutic strategies against *F. nucleatum* in the context of UC, hoping to provide innovative approaches to treatment. The causal relationship between *F. nucleatum* and UC remains elusive, but the persistent stimulation of the UC gut by *F. nucleatum* exacerbates the disease’s progression and potentially elevates the risk of cancer. Therefore, a deeper understanding of the mechanisms behind the dysbiosis of the gut flora, involving *F. nucleatum* and UC, may form the foundation for preventing or treating UC and other intestinal inflammatory diseases. Evaluating the regulation of microbial communities at both ends of the oral-gut axis could become a beneficial protective factor for intestinal diseases, and oral health should continue to be a focal point for UC patients. Utilizing probiotics, prebiotics, or diet-related treatments may emerge as a potentially fruitful area of research for future UC therapies. This approach is of significant importance for further exploration of the role of *F. nucleatum* in UC.To bridge these knowledge gaps, a structured research agenda integrating mechanistic exploration and translational innovation is proposed in the following section.

## 6 Future research directions

**Figure 2:**
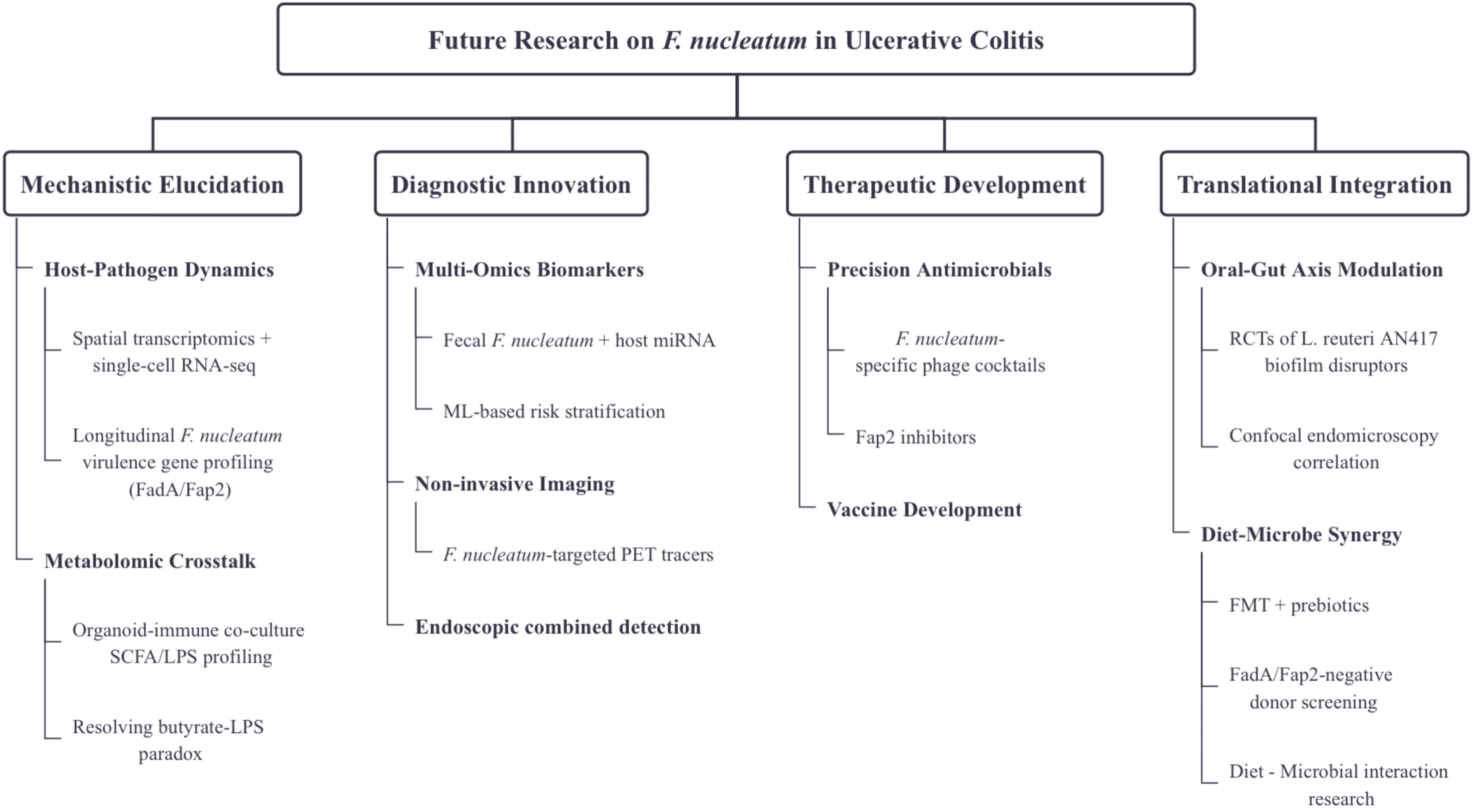
Roadmap for future research on *F. nucleatum* in UC.

While this review synthesizes current evidence on *F. nucleatum* in UC, critical knowledge gaps remain. To advance clinical translation, we propose the following prioritized research axes (**Figure 2**).

### 6.1 Mechanistic elucidation

Host-Pathogen Dynamics: Employ spatial transcriptomics and single-cell RNA sequencing to map *F. nucleatum*-epithelial/immune cell interactions across UC disease stages. Prioritize longitudinal sampling to capture temporal shifts in *F. nucleatum* virulence gene expression (e.g., FadA, Fap2) during flare vs remission.

Metabolomic Crosstalk: Systematically profile Fn-derived metabolites (e.g., butyrate, LPS) and their immunomodulatory effects using organoid-immune co-culture models. Investigate the specific molecular mechanisms of OMVs and FnEVs in gut barrier disruption. Focus on SCFA paradox: reconcile *F. nucleatum*’s anti-inflammatory butyrate production with its pro-inflammatory LPS.

### 6.2 Diagnostic innovation

Multi-Omics Biomarkers: Develop non-invasive diagnostic tools based on F. nucleatum virulence genes (e.g., FadA, Fap2). Validate fecal OMVs and FnEVs as biomarkers for mucosal healing and therapeutic response, and integrate multi-omics data (e.g., microbiome, metabolomics, miRNA) to establish predictive models.

Non-invasive Imaging: Develop *F. nucleatum*-targeted PET tracers to visualize *F. nucleatum* colonization in UC-CRC transition.

Endoscopic combined detection: Integrate confocal laser endomicroscopy with OMV level analysis to assess the correlation between mucosal healing and therapeutic response.

### 6.3 Therapeutic development

Precision Antimicrobials: Optimize *F. nucleatum*-specific phage cocktails targeting RadD-mediated biofilms. Conduct structure-activity studies on Fap2 inhibitors to block hematogenous dissemination.

Vaccine Development: Advance clinical trials of FadA/Fap2-based subunit vaccines to evaluate their anti-inflammatory effects in UC.

### 6.4 Translational integration

Oral-Gut Axis Modulation: Launch RCTs testing periodontal interventions (e.g., L. reuteri AN417 biofilm disruptors) on UC outcomes. Correlate oral *F. nucleatum* reduction with intestinal barrier restoration via confocal endomicroscopy.

Diet-Microbe Synergy: Explore FMT combined with prebiotics (e.g., fucoidan) to competitively exclude *F. nucleatum* Standardize donor screening for FadA/Fap2 gene absence.Conduct dietary intervention trials to determine the impact of dietary fibers (such as fructooligosaccharides) on *F. nucleatum* colonization.
